# A Genetic Variant in *CD274* Is Associated With Prognosis in Metastatic Colorectal Cancer Patients Treated With Bevacizumab-Based Chemotherapy

**DOI:** 10.3389/fonc.2022.922342

**Published:** 2022-06-28

**Authors:** Wan Qin, Ben Zhao, Duanrui Wang, Jiamin Liu, Yilu Zhou, Wenjun Zhu, Yongbiao Huang, Hong Qiu, Xianglin Yuan

**Affiliations:** ^1^ Department of Oncology, Tongji Hospital, Huazhong University of Science and Technology, Wuhan, China; ^2^ Department of Cancer Biotherapy Center, The Third Affiliated Hospital of Kunming Medical University (Tumor Hospital of Yunnan Province), Kunming, China; ^3^ Biological Sciences, Faculty of Environmental and Life Sciences, University of Southampton, Southampton, United Kingdom; ^4^ Institute for Life Sciences, University of Southampton, Southampton, United Kingdom

**Keywords:** metastatic colorectal cancer, bevacizumab, single-nucleotide polymorphisms, *CD274*, prognosis

## Abstract

Bevacizumab plus chemotherapy is a well-established first-line treatment for metastatic colorectal cancer (mCRC). We investigated whether polymorphisms of genes involved in immune regulation signaling are related to the clinical outcome of mCRC patients treated with bevacizumab-based chemotherapy. In this study, we genotyped 14 single-nucleotide polymorphisms (SNP) in IFN-γ/IFNGRs/JAKs/STATs/PD-L1 pathway by using DNA from blood samples of 141 mCRC patients treated with first-line bevacizumab-based chemotherapy. In the univariate and multivariate analysis, patients with AA genotype of *CD274*:rs2297136 had a significantly better PFS and OS than patients with AG or GG genotype (10.8 versus 9.8, log-rank *P*=0.0031; 31.4 versus 20.9, log-rank *P*=0.0233). Patients with AG/GG genotype of *IFNGR1*:rs2234711, CT/TT genotype of *IFNGR1*:rs9376267 also showed longer OS than patients with AA or CC genotype, however, the statistic did not reach significant after adjusted by clinical factors in the multivariate analysis. A nomogram based on the genetic variants and clinic characteristics was developed with a good accuracy to predict patients’ survival. Our result indicates that *CD274*:rs2297136 is one of the most important predictors for the prognosis of mCRC patients treated with bevacizumab-based chemotherapy, if further validated in larger population.

## Introduction

Colorectal cancer (CRC) is the fourth most commonly diagnosed cancer and ranks second in mortality rates with both sexes combined in the world (1). CRC is a curable disease if diagnosed in early stages; however, between 70 and 90% of CRC cases are currently diagnosed in advanced stages of the disease, resulting in fluoropyrimidine-based chemotherapy as the main treatment option. Bevacizumab, a humanized monoclonal antibody that targets VEGF, was first approved for metastatic colorectal cancer by Food and Drug Administration (FDA) in 2004 (2, 3). From then on, bevacizumab has shown benefits to mCRC patients as first-line treatment in association with standard chemotherapy (4). In many cases, however, clinical outcomes can be highly variable, with some patients responding remarkably well and others not. The identification of who will obtain the greater benefit from this therapy is still a major question for clinicians.

Several studies have reported the prognostic and predictive values of some factors such as plasma VEGF concentration (5), circulating tumor cells (6), and tumor VEGF expression (7) in colorectal cancer. However, these single biomarkers are yet to be replicated in clinical contexts. It is needed to seek more effective indicators for early prognosis prediction for these patients. Recently, germline polymorphisms of genes which function in various fundamental cellular process are proved to determine the prognosis of patients receiving bevacizumab-contained chemotherapy (8). For example, VEGF -1498 CC and VEGF-1154 GA+AA genotype have been reported to be associated with better prognosis of patients treated with bevacizumab-based chemotherapy (9, 10). Besides, SNPs in genes functioning in vitamin D transport (11), cancer stem cells (12), MAPK signaling pathway (13) also have been demonstrated to possess intriguing links with bevacizumab treatment efficacy.

During the past two decades, the host immune system has been demonstrated to play an important role in killing cancer cells after chemotherapy or anti-angiogenic agent treatment. Beyond the cytotoxic properties, conventional anti-neoplastic agents as well as anti-VEGF therapy also have the ability to stimulate the innate and acquired immune system, and in some instances even to evoke long-term protective memory T cell responses, thus facilitating tumor eradication (14). IFN-γ is an important endogenous regulator of immune responses and plays crucial roles in eliminating tumor cells through direct induction of cytotoxic activities as well as enhancing the Th1-related immune response (15). Extracellular IFN-γ signals through two transmembrane receptors, namely IFNGR1 and IFNGR2, and activates receptors-associated JAKs, which results in phosphorylation of STATs and translocation to the nucleus to bind the IFN-γ target genes (16). IFN-γ was reported as the most potent inducer of PD-L1 expression which acted mainly through classical JAK-STAT pathway (17–19). Nikolaos et al. reported that the serum IFN-γ was significantly increased in chemotherapy plus bevacizumab group compared to chemotherapy alone group after 2 cycle treatment in mCRC patients (20). Moreover, in a preclinical study, Bevacizumab could stimulate GD2-CAR T cells to produce IFN-γ to kill neuroblastoma (21). We speculate that polymorphisms of genes in IFN-γ/IFNGRs/JAKs/STATs/PD-L1 pathway may affect the antitumor immune activity, thereby influencing clinical outcomes of bevacizumab-based chemotherapy in patients with mCRC.

In the current study, we evaluated the association of genetic polymorphisms in IFN-γ/IFNGRs/JAKs/STATs/PD-L1 pathway with the clinical outcome of mCRC patients undergoing first-line bevacizumab-based chemotherapy.

## Materials and Methods

### Study Population

Patients with a pathologically and radiologically confirmed metastatic colorectal adenocarcinoma were included. They all received first-line oxaliplatin- or irinotecan-based chemotherapy plus bevacizumab at Tongji Hospital, Huazhong University of Science and Technology (Wuhan, Hubei Province, China) between 2013 and 2019. Patients with prior adjuvant chemotherapy with no prior treatment for metastatic disease nor exposure to bevacizumab were also included if more than 12 months had elapsed between the end of treatment and the diagnosis of metastasis. Progression free survival (PFS) was calculated from the date of mCRC diagnosis to the date of disease progression or the date of censoring of live cases, whichever came first. Overall survival (OS) was calculated from the date of mCRC diagnosis to the date of death or the date of censoring of live cases, whichever came first.

Of the 168 patients eligible for this study, 16 were excluded because of insufficient DNA for genotyping, 11 because of incomplete data on medical record, leaving 141 patients with complete information for the current analysis. The study was approved by the Ethics Committee of Tongji Medical College, Huazhong University of Science and Technology. Written informed consent was obtained from all patients before interview.

### Genotyping Methods

Blood samples were collected and genomic DNA was extracted with a PureLink Genomic DNA Mini Kit (Invitrogen, K1820-01 Invitrogen, Waltham, MA, USA) according to the manufacturer’s protocol, and stored at -80°C until use. SNPs in 8 core genes in immune activity (*IFNG, IFNGR1, IFNGR2, JAK1, JAK2, STAT1, STAT2* and *CD274*) were screened in Ensemble database (http://asia.ensembl.org/index.html) and NCBI website (https://www.ncbi.nlm.nih.gov/snp/). 14 SNPs were identified with a minor allele frequency >5% in the Chinese population and linkage disequilibrium (LD) analysis with a r^2^<0.80 (**Table 1**). SNPs were genotyped in Capitalbio Technology Corporation (Beijing, China) by using MALDI-TOF mass spectrophotometry to detect allele-specific primer extension products with the MassARRAY platform (Sequenom, Inc.).

**Table 1:** Genes and SNPs selected for analysis.

Gene	SNP	Allelic change	Functional consequence
*IFNG*	rs2069718	A/G	intron variant
	rs1861493	C/T	intron variant
*IFNGR1*	rs2234711	A/G	5 prime UTR variant
	rs9376267	C/T	5 prime UTR variant
*IFNGR2*	rs9808753	A/G	missense variant
	rs1059293	C/T	3 prime UTR variant
*JAK1*	rs112395617	DEL.AATT/AATT	3 prime UTR variant
*JAK2*	rs1887429	G/T	5 prime UTR variant
	rs1887428	G/C/T	5 prime UTR variant
*STAT1*	rs3088307	C/G	3 prime UTR variant
	rs41430444	T/C	5 prime UTR variant
	rs6745710	C/G	5 prime UTR variant
*STAT2*	rs2020854	T/C	Splice region variant
*CD274(PD-L1)*	rs2297136	G/A	3 prime UTR variant

### Bioinformatic Analysis

We used the Genotype-Tissue Expression (GTEx, http://www.gtexportal.org/home/) portal to identify potential associations between genetic variants and gene expression levels (eQTL) in all available tissues (22). The correlation of genetic variants of *CD274*:rs2297136 and *CD274* expression in whole blood tissues was calculated using the R programming language version 3.5.1.

The genetic variant and the clinical parameters were included in the development of the predictive nomogram to predict overall survival of mCRC patients *via* the “rms” R package. Kaplan-Meier survival curve was generated by the “survminer” R package. ROC curve was plotted by using “timeROC” R package.

### Statistical Analysis

Statistical analyses were conducted by Graphpad Prism V8.2 or R software 3.6.3. Survival data were analyzed using the Kaplan-Meier method, and compared using log-rank test. Cox proportional hazard analysis was applied to estimate the hazard ratio (HR) and 95% confidence intervals (CIs) of all factors possibly related to the prognosis of patients. Multivariate Cox regression analysis was used for the adjustment of covariates. A *P* value <0.05 was considered to be statistically significant.

## Results

### Patient Characteristics and Clinical Outcome

Clinicopathologic characteristics of 141 patients enrolled in this study are present in **Table 2**. This cohort included 71 males (50.4%) and 70 females (49.6%) with a median age of 52.0 years (range, 24–80 years). The median follow-up time was 36.9 months, with 83 deaths (58.9%) observed. The median PFS and OS was 10.6 and 28.5 months, respectively.

**Table 2:** Patient characteristics (N=141).

Variables		N		Percentage (%)
Gender				
	Male	71		50.4%
	Female	70		49.6%
Age				
	<65	127		90.1%
	≥65	14		9.9%
KPS				
	80-100	115		81.6%
	<80	26		18.4%
Primary tumor site				
	Right side	35		24.8%
	Left side	106		75.2%
Liver only metastasis				
	Yes	25		17.7%
	No	116		82.3%
Number of metastases				
	1	39		27.7%
	>1	102		72.3%
Time to metastasis				
	Synchronous		79	56.0%
	Metachronous		62	44.0%
Chemotherapy				
	Oxaliplatin-based regimen		76	53.9%
	Irinotecan-based regimen		65	46.1%
*KRAS* status				
	Mutant	36		25.5%
	Wildtype	34		24.1%
	Unknown	71		50.4%

Associations between baseline characteristics and clinical outcomes were examined using the log-rank test in univariate analysis (**Supplementary Table 1**). We found that patients with metachronous tumors had a significantly longer PFS and OS compared with patients with synchronous tumors (13.16 versus 9.3 months, HR= 1.95, 95%CI=1.3~2.92, *P* = 0.001; 34.0 versus 20.93 months, HR= 0.43, 95%CI= 0.27~0.68, *P*<0.001, respectively). In addition, patients with KPS under 80 had a shorter OS compared with patients with KPS≥80 (20.93 versus 31.4 months, HR= 1.74, 95%CI=1.06~2.88, *P* = 0.03).

### Clinical Outcomes of Genetic Variants in IFN-γ/IFNGRs/JAKs/STATs/PD-L1 Pathway in mCRC Patients Receiving Bevacizumab-Based Chemotherapy

**Figure 1:**
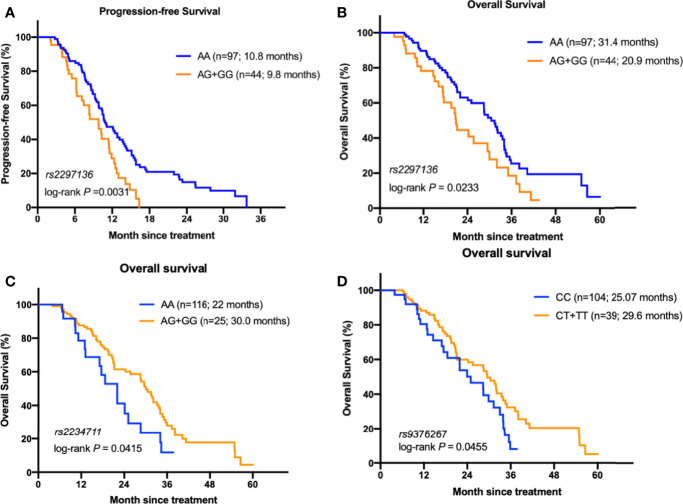
The relationship between the genetic variants with the prognosis of mCRC patients receiving bevacizumab-based chemotherapy. **(A)** Kaplan–Meier curves of PFS stratified by *CD274*: rs2297136 genotype. **(B)** Kaplan–Meier curves of OS stratified by *CD274*: rs2297136 genotype. **(C)** Kaplan–Meier curves of OS stratified by *IFNGR1*: rs2234711 genotype. **(D)** Kaplan–Meier curves of OS stratified by *IFNGR1*: rs9376267 genotype.

We assessed potential association of each of the 14 individual SNP with clinical outcome of mCRC patients. In the Kaplan-Meier estimates, patients with AA genotype of *CD274*:rs2297136 had a significantly better PFS and OS than patients with AG or GG genotype (10.8 versus 9.8, HR=1.79, 95%CI=1.23~2.83, *P*=0.0031; 31.4 versus 20.9, HR= 1.73, 95%CI=1.05~2.88, *P*=0.0233, **Figures 1A, B**). In the multivariable analysis, the association between genotype of *CD274*:rs2297136 and clinical outcome remained significant after adjusted by KPS and Time to metastasis (HR= 1.68, 95%CI=1.09~2.59, *P*= 0.018 for PFS; HR= 1.88, 95%CI=1.15~3.08, *P*= 0.012 for OS, **Table 3**).

**Table 3:** Association between genotypes and clinical outcome of mCRC patients treated with bevacizumab-based chemotherapy.

Progression-Free Survival				Overall Survival			
Genotypes	N	Median, months(95%CI)	HR (95%CI)	*P* value*	Median, months(95%CI)	HR (95%CI)	*P* value*
*CD274*:rs2297136				0.018			0.012
AA	97	10.8 (9.18,12.42)	1		31.4 (27.74,35.06)	1	
AG+GG	44	9.8 (7.14,12.46)	1.68 (1.09,2.59)		20.90 (18.80,23.00)	1.88 (1.15,3.08)	
*IFNGR1*:rs2234711				0.845			0.237
AA	26	10.33 (9.43,11.24)	1		22.0 (16.24,27.76)	1	
AG+GG	115	10.67 (9.09,12.10)	0.93 (0.43,1.997)		30.0 (26.81,33.19)	0.61 (0.27,1.38)	
*IFNGR1*:rs9376267				0.967			0.239
CC	38	10.8 (9.78,11.82)	1		25.07 (15.30,34.84)	1	
CT+TT	103	10.6 (9.07,12.13)	1.01 (0.54,1.90)		30.5 (27.18,33.82)	0.66 (0.33,1.32)	

*P value was calculated by multivariate analyses adjusted for KPS and Time to metastasis.

In the *KRAS* mutant subgroup, patients with AA genotype of rs2297136 had a significantly longer PFS than patients with any G genotype (P =0.04, **Supplementary Figure 1A**). However, due to the limited number of patients, the difference didn’t reach significance for OS (P=0.09, **Supplementary Figure 1B**).

Patients with AG/GG genotype of *IFNGR1*:rs2234711, CT/TT genotype of *IFNGR1*:rs9376267 also showed much longer OS than patients with AA or CC genotype (30.0 versus 22.0 months, *P*=0.041, **Figure 1C**; 30.5 versus 25.07 months, *P*=0.0312, **Figure 1D**). However, the statistic did not reach significant after adjusted by KPS and Time to metastasis in the multivariate analysis (*P* = 0.237 and 0.239, respectively, **Table 3**).

Similar analyses of the other 11 SNPs showed no associations between genotype and clinical outcome in mCRC patients (**Supplementary Table 2**).

### Functional Effect

**Figure 2:**
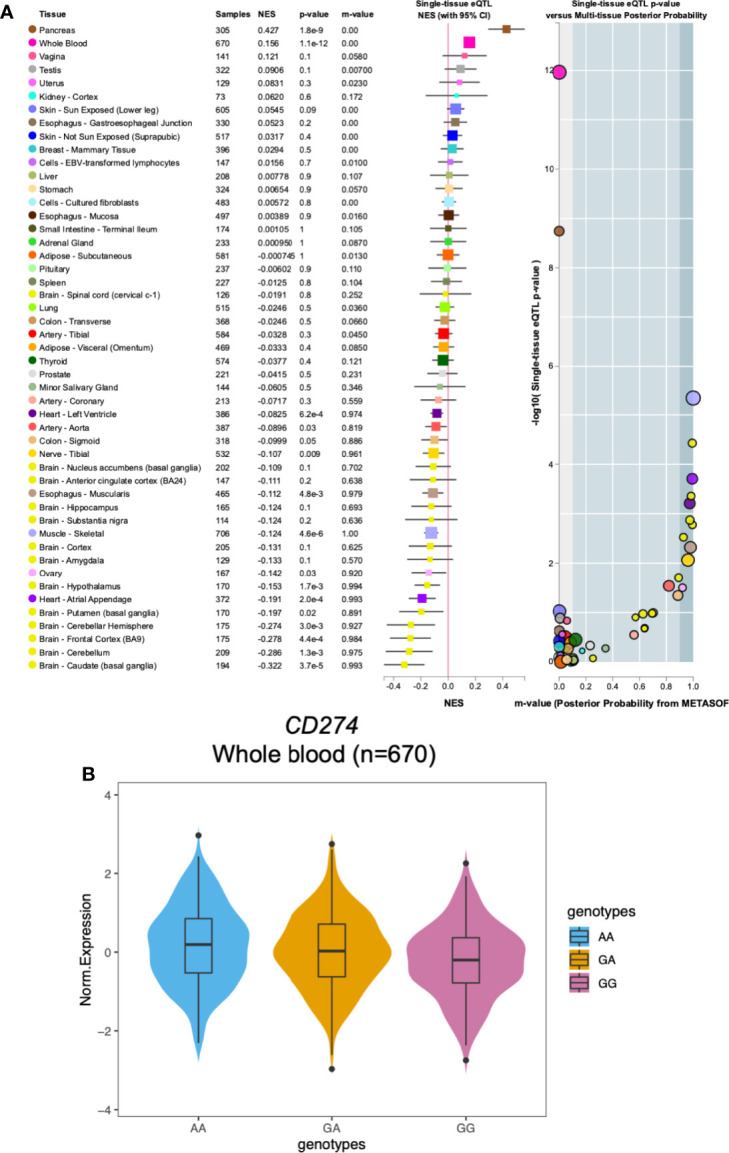
The relationship between the *CD274*: rs2297136 genotype with gene expression in Genotype-Tissue Expression (GTEx) dataset. **(A)** The association of *CD274*: rs2297136 genotype with gene expression in multiple tissues in GTEx dataset. **(B)** The association of *CD274*: rs2297136 genotype with gene expression in 670 whole blood samples in GTEx dataset.

We utilized data from Genotype-Tissue Expression (GTEx) dataset to provide possible explanations for our finding that *CD274*: rs2297136 was associated with PFS and OS of mCRC patients treated with bevacizumab-based chemotherapy. The association of *CD274*: rs2297136 with the normalized mRNA expression of *CD274* in multiple tissues is shown in **Figure 2A**. Interestingly, we found that in the 670 whole blood samples, AA genotype of *CD274*: rs2297136 was associated with an increased mRNA expression of the *CD274* gene compared with the GA and GG genotypes (**Figure 2B**). The association between the genotype of rs2297136 and *CD274* expression was significant with a *P* value of 1.1e-12.

### A Predictive Nomogram Development and Validation

**Figure 3:**
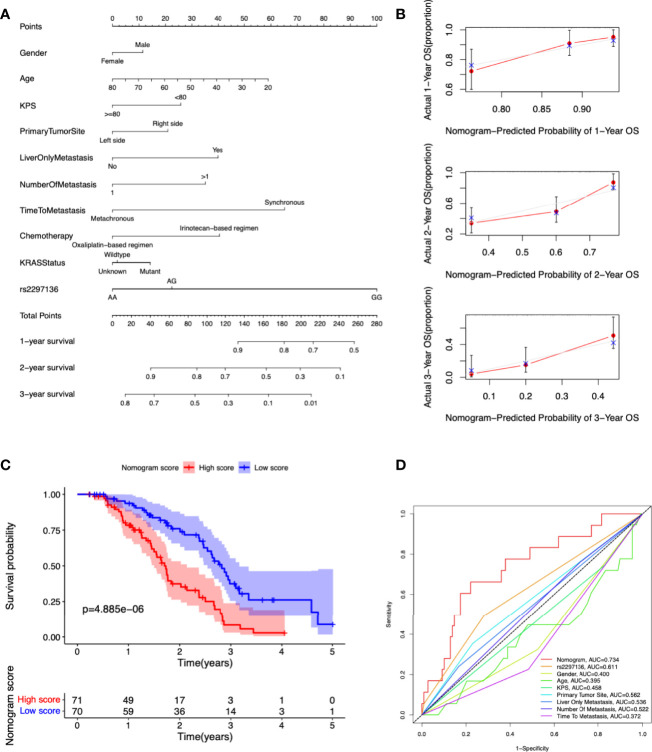
Development and validation a nomogram for predicting prognosis of mCRC patients treated with bevacizumab-based chemotherapy. **(A)** A nomogram was constructed to predict 1-, 2-, 3-year OS. **(B)** Calibration plots of the nomogram for 1-, 2-, 3-year OS. **(C)** Kaplan-Meier curves of OS based on the nomogram scores. **(D)** The ROC curves of nomogram score, rs2297136 and other clinical factors.

In order to provide a useful clinical application of our findings, we constructed a nomogram for calculating the 1-, 2-, 3-year OS rate by using the genetic variants and clinic information included in our study (**Figure 3A**). Calibration curves at 1, 2 and 3 years indicated that the nomogram could accurately predict OS (**Figure 3B**). Patients were segregated into high-score and low-score groups determined by the median nomogram score, and Kaplan-Meier analysis indicated that the patients with low nomogram score had significantly better overall survival (*P*=4.885e-06) (**Figure 3C**). The AUCs of the nomogram in the 1-year ROC curves were 0.734, which outperformed the rs2297136 and other clinical factors (**Figure 3D**).

## Discussion

Accumulating evidence indicates that conventional chemotherapy and antiangiogenic agents not only exert direct cytostatic or cytotoxic effects, but also activate tumor-targeting immune responses (23, 24). Altered immune checkpoint function may therefore influence the anti-cancer treatment outcomes. In this study, we determined whether genetic variations in the IFN-γ/IFNGRs/JAKs/STATs/PD-L1 pathway were associated with prognosis of late-stage CRC patients receiving bevacizumab-based chemotherapy. We found that AA genotype of *CD274*:rs2297136 had a significantly longer median PFS and OS than patients with any G allele. Notably, the difference in OS (9.6 months) was more prominent than PFS (1.0 months), which implied that the polymorphism tended to predict the prognosis of these patients, rather than the therapeutic effect to bevacizumab -based chemotherapy.

*CD274* is located in 9p24.1. It consists of 7 exons and is approximately 20 kb in size. This gene encodes PD-L1, which is an immune inhibitory receptor ligand that is expressed by hematopoietic and non-hematopoietic cells, such as T cells and B cells and various types of tumor cells (25). Hu et al. reported that in advanced non-small cell lung cancer (NSCLC) patients receiving apatinib treatment, patients with TT(AA) genotype of *CD274*:rs2297136 had a significantly longer median PFS and OS than patients with any C(G) allele (26). As apatinib is also an antiangiogenic drug, our study and theirs both support the link between AA genotype of *CD274*:rs2297136 and a favor response to antiangiogenic therapy. In other studies, this SNP has been reported to be correlated with response to cancer therapy. For example, in a study evaluating the relationship between polymorphisms in immune regulatory pathways with cetuximab efficacy in mCRC patients, *CD274*:rs2297136 genetic variant showed impact on the response to cetuximab (27). In a cohort of NSCLC patients treated with first-line paclitaxel-cisplatin chemotherapy, genetic variant of *CD274* was reported to be associated with prognosis of patients (28). In a study conducted by Xie et al., *CD274*:rs2297136 was associated with the risk and overall survival of hepatocellular carcinoma (29).

PD-L1 expression is prognostic in many types of human malignancies, including CRC. In an integrated meta-analysis including twelve studies of 4344 patients, high expression of PD-L1 in the tumor was correlated with poor OS in colorectal cancer (30). In these early studies, however, the focus was on tumor cells rather than on tumor-infiltrating immune cells (TIICs). Recent studies revealed that in contrast to the impact of PD-L1-expressing tumor cells, high expression of PD-L1 on TIICs was a marker of good prognosis (31). In line with this, Berntsson J *et al.* reported that high PD-L1 expression on TIICs was an independent factor of a prolonged OS in colon cancer (32). In the 670 whole blood samples from the GTEx database, AA genotype of *CD274*: rs2297136 is associated with a significantly increased mRNA expression of the *CD274* gene compared with any G allele. Combining our experimental and literature data, we speculate that patients with AA genotype of *CD274*: rs2297136 may correlate to high PD-L1 expression on TIICs and better prognosis to anti-VEGF therapy in mCRC patients.

In our study, the other two polymorphisms associated with the OS of mCRC patients were in *IFNGR1*. This gene encodes a ligand-binding chain, and forms the heterodimer receptor for the cytokine IFN-γ with IFNGR2 (33). Genetic variation in *IFNGR1* is associated with susceptibility to pulmonary tuberculosis (34, 35), Helicobacter pylori (36) and early gastric carcinoma (37). We found that patients with AG/GG genotype of *IFNGR1*:rs2234711, CT/TT genotype of *IFNGR1*:rs9376267 had longer OS than patients with AA or CC genotype. Both variations are in the promoter region of *IFNGR1*. Although the difference did not reach statistic significant by multivariable analysis in our limited number of samples, our findings support further investigation in a larger population.

There are some limitations in our study. Firstly, as a comparison cohort not treated with bevacizumab is not available, whether the polymorphism could predict the therapeutic effect to bevacizumab is still unknown and needs to be validated in further studies. Secondly, as the results are based on an analysis of limited number of patients, larger samples and replication analyses in other cohorts are required to confirm the findings. Thirdly, in our study, *KRAS* mutation status was only detected in half of the patients. Other clinical factors, such as *BRAF* mutation and microsatellite status, were not assayed in our cohorts. The relationship between these factors with the polymorphism and their correlation with prognosis is still unknown. Last but not least, the underlying molecular mechanisms of the genetic variant of *CD274*:rs2297136 have not be examined in this study, and remain to be studied in the future.

In summary, we found genetic variant of *CD274*:rs2297136 was related to the clinical outcome of metastatic colorectal cancer (mCRC) patients treated with bevacizumab-based chemotherapy. We also constructed a novel predictive nomogram by integrating the polymorphism with several clinical characteristics for convenient clinical application. In the future, moving from conventional treatment to precision medicine, the verification and validation of existing SNPs as well as the identification of newer ones is anticipated to greatly impact on the management of mCRC.

## Data Availability Statement

The datasets presented in this study can be found in online repositories. The names of the repository/repositories and accession number(s) can be found in the article/**Supplementary Material**.

## Ethics Statement

The studies involving human participants were reviewed and approved by the Ethics Committee of Tongji Medical College, Huazhong University of Science and Technology. The patients/participants provided their written informed consent to participate in this study.

## Author Contributions

WQ analyzed the data and wrote the manuscript. WQ, BZ, DW, JL, WZ, YH collected the blood samples and clinical information. YZ participated the bioinformatic analysis. HQ and XY designed the study and modified the manuscript. All authors have read and approved the manuscript for publication. All authors contributed to the article and approved the submitted version.

## Funding

This study was supported by the State Key Program of National Natural Science Foundation of China (Grant No.82130092) and the Radiotherapy and Protection Engineering Center Innovation Capacity Building Project of Hubei Province (Grant No.2018-420114-35-03-071705).

## Conflict of Interest

The authors declare that the research was conducted in the absence of any commercial or financial relationships that could be construed as a potential conflict of interest.

## Publisher’s Note

All claims expressed in this article are solely those of the authors and do not necessarily represent those of their affiliated organizations, or those of the publisher, the editors and the reviewers. Any product that may be evaluated in this article, or claim that may be made by its manufacturer, is not guaranteed or endorsed by the publisher.

## Supplementary Material

Supplementary Figure 1 The relationship between the genetic variants with the prognosis of mCRC patients with *KRAS* mutant. (a) Kaplan–Meier curves of PFS stratified by *CD274*: rs2297136 genotype. (b) Kaplan–Meier curves of OS stratified by *CD274*: rs2297136 genotype.
