# Supplementary figures and images for: A Genetic Variant in CD274 Is Associated With Prognosis in Metastatic Colorectal Cancer Patients Treated With Bevacizumab-Based Chemotherapy
Source: Front Oncol. 2022 Jun 28;12:922342. doi: 10.3389/fonc.2022.922342 (PMC9275392; doi:10.3389/fonc.2022.922342)

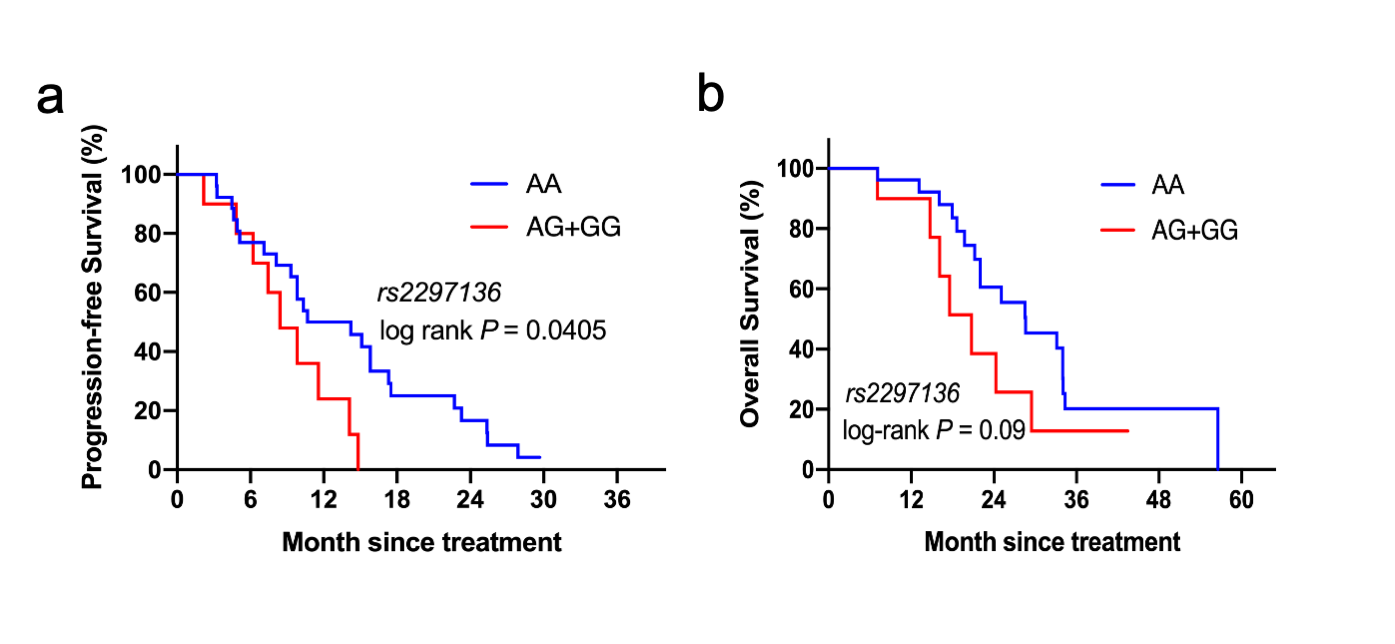

Supplement: Supplementary Figure 1 — The relationship between the genetic variants with the prognosis of mCRC patients with KRAS mutant. (a) Kaplan–Meier curves of PFS stratified by CD274: rs2297136 genotype. (b) Kaplan–Meier curves of OS stratified by CD274: rs2297136 genotype. [file Image_1.tiff]
