# Supplementary material for: A Genetic Variant in CD274 Is Associated With Prognosis in Metastatic Colorectal Cancer Patients Treated With Bevacizumab-Based Chemotherapy
Source: Front Oncol. 2022 Jun 28;12:922342. doi: 10.3389/fonc.2022.922342 (PMC9275392; doi:10.3389/fonc.2022.922342)
Supplement: Supplementary file 2 [file Table_1.docx]

| Table S1: Patient characteristics and association with clinical outcomes. | | | | |  |  |  |  |
| --- | --- | --- | --- | --- | --- | --- | --- | --- |
|  |  |  | Progression-Free Survival | | | Overall Survival | | |
| Variables |  | N | Median, months (95%CI) | HR (95%CI) | *P* value^†^ | Median, months (95%CI) | HR (95%CI) | *P* value^†^ |
| Gender |  |  |  |  | 0.576 |  |  | 0.24 |
|  | Male | 71 | 10.67(9.25,12.09) | 1 |  | 30.0(22.49,37.51) | 1 |  |
|  | Female | 70 | 10.60(9.17,12.03) | 0.90(0.62,1.31) |  | 28.53(22.51,34.56) | 0.77(0.50,1.19) |  |
| Age |  |  |  |  | 0.867 |  |  | 0.688 |
|  | <65 | 127 | 10.43(9.3,11.57) | 1 |  | 28.53(23.65,33.41) | 1 |  |
|  | ≥65 | 14 | 11.57(8.27,14.87) | 0.95(0.53,1.70) |  | 32.07(6.94,57.19) | 0.87(0.45,1.70) |  |
| KPS |  |  |  |  | 0.292 |  |  | **0.03** |
|  | 80-100 | 115 | 10.8(9.42,12.18) | 1 |  | 31.4(27.04,35.76) | 1 |  |
|  | <80 | 26 | 9.5(7.38,11.62) | 1.30(0.80,2.12) |  | 20.93(12.17,29.7) | 1.74(1.06,2.88) |  |
| Primary tumor site | |  |  |  | 0.713 |  |  |  |
|  | Right side | 35 | 10.23(8.0,12.46) | 1 |  | 29.47(20.28,38.65) | 1 | 0.788 |
|  | Left side | 106 | 10.67(9.5,11.83) | 0.92(0.58,1.46) |  | 28.63(22.74,34.53) | 1.08(0.63,1.84) |  |
| Liver only metastasis | |  |  |  | 0.607 |  |  | 0.722 |
|  | Yes | 25 | 10.3(9.11,11.48) | 1 |  | 28.47(19.21,37.73) | 1 |  |
|  | No | 116 | 10.6(9.0,12.20) | 0.88(0.54, 1.43) |  | 29.6(23.18,36.02) | 1.13(0.58,2.21) |  |
| Number of metastases | |  |  |  |  |  |  | 0.096 |
|  | 1 | 39 | 12.8(9.10,16.5) | 1 | 0.169 | 31.4(26.44,36.36) | 1 |  |
|  | ＞1 | 102 | 10.33(9.11,11.56) | 1.35(0.88,2.08) |  | 28.63(22.95,34.32) | 1.63(0.92,2.91) |  |
| Time to metastasis | |  |  |  | **0.001** |  |  | **<0.001** |
|  | Synchronous | 78 | 9.3(8.14,10.46) | 1 |  | 20.93(19.17,22.70) | 1 |  |
|  | Metachronous | 63 | 13.16(10.88,15.46) | 0.43(0.27,0.68) |  | 34.00(27.88,40.12) | 0.43(0.27,0.68) |  |
| Chemotherapy | |  |  |  | 0.626 |  |  | 0.335 |
| Oxaliplatin-based  Irinotecan-based | | 76 | 10.43(8.66,12.21) | 1 |  | 31.6(28.04,35.23) | 1 |  |
|  |  | 65 | 10.67(9.30,12.03) | 0.91(0.62,1.33) |  | 25.07(17.31,32.82) | 1.24(0.80,1.91) |  |
| *KRAS* status | |  |  |  | 0.74 |  |  | 0.892 |
|  | Mutant | 36 | 10.33(8.2, 12.49) | 1 |  | 25.07(14.36,35.77) | 1 |  |
|  | Wildtype | 34 | 10.3(8.49,12.11) | 1.19(0.70,2.04) |  | 31.4(13.03,49.77) | 1.03(0.56,1.91) |  |
|  | Unknown | 71 | 11.4(9.48, 13.32) | 0.99(0.64,1.53) |  | 30.5(26.47,34.53) | 0.91(0.55,1.50) |  |

CI, confidence interval; HR, hazard ratio; KPS, Karnofsky Performance Status

† *P* value was based on log-rank test for PFS and OS in the univariate analysis.
