# Supplementary material for: A Genetic Variant in CD274 Is Associated With Prognosis in Metastatic Colorectal Cancer Patients Treated With Bevacizumab-Based Chemotherapy
Source: Front Oncol. 2022 Jun 28;12:922342. doi: 10.3389/fonc.2022.922342 (PMC9275392; doi:10.3389/fonc.2022.922342)
Supplement: Supplementary file 3 [file Table_2.docx]

| Table S2: Association between SNP with clinical outcomes. | | | |  |  |  |  |
| --- | --- | --- | --- | --- | --- | --- | --- |
|  |  | Progression-Free Survival | | | Overall Survival | | |
|  | N | Median, months (95%CI) | HR (95%CI) | *P* value^†^ | Median, months (95%CI) | HR (95%CI) | *P* value^†^ |
| rs2069718 |  |  |  | 0.4538 |  |  | 0.717 |
| AA | 105 | 10.67(9.18,12.16) | 1 |  | 28.5(22.99,34.01) | 1 |  |
| AG+GG | 36 | 10.43(8.78,12.09) | 1.18(0.75,1.86) |  | 32.07(27.09,37.05) | 1.10(0.66,1.82) |  |
| rs1861493 |  |  |  | 0.75 |  |  | 0.801 |
| CC | 31 | 10.8(7.67,13.93) | 1 |  | 28.63(14.00,43.27) | 1 |  |
| CT+TT | 110 | 10.43(9.41,11.46) | 1.07(0.69,1.66) |  | 29.47(24.18,34.75) | 1.07(0.64,1.78) |  |
| rs2234711 |  |  |  | 0.82 |  |  | **0.041** |
| AA | 26 | 10.33(9.43,11.24) | 1 |  | 22.0(16.24,27.76) | 1 |  |
| AG+GG | 115 | 10.67(9.09,12.10) | 1.06(0.64,1.75) |  | 30.0(26.81,33.19) | 0.91(0.58,1.44) |  |
| rs9376267 |  |  |  | 0.95 |  |  | **0.0312** |
| CC | 38 | 10.8(9.78,11.82) | 1 |  | 25.07(15.30,34.84) | 1 |  |
| CT+TT | 103 | 10.6(9.07,12.13) | 0.99(0.65,1.51) |  | 30.5(27.18,33.82) | 0.61(0.37,1.02) |  |
| rs9808753 |  |  |  | 0.367 |  |  | 0.176 |
| AA | 52 | 10.6(8.37,12.83) | 1 |  | 30.0(22.82,37.18) | 1 |  |
| AG+GG | 89 | 10.6(9.47,11.73) | 1.19(0.81,1.75) |  | 28.47(20.78,36.16) | 1.44(0.94,2.22) |  |
| rs1059293 |  |  |  | 0.389 |  |  | 0.735 |
| TT | 103 | 11.0(9.57,12.43) | 1 |  | 28.63(23.85,33.42) | 1 |  |
| CT+CC | 38 | 8.87(7.0,10.73) | 1.21(0.76,1.91) |  | 28.53(15.60,41.47) | 1.12(0.69,1.80) |  |
| rs112395617 |  |  |  | 0.66 |  |  | 0.782 |
| AATT | 52 | 10.43(9.16,11.71) | 1 |  | 31.63(27.30,35.97) | 1 |  |
| DEL.AATT | 89 | 10.6(9.39,11.81) | 0.92(0.63,1.35) |  | 28.5(19.13,37.87) | 0.90(0.57,1.41) |  |
| rs1887429 |  |  |  | 0.0562 |  |  | 0.222 |
| GG | 97 | 11.1(8.83,13.37) | 1 |  | 29.47(23.75,35.19) | 1 |  |
| GT+TT | 44 | 10.23(8.32,12.14) | 1.49(0.96,2.32) |  | 25.07(16.13,34.00) | 1.39(0.85,2.28) |  |
| rs1887428 |  |  |  | 0.452 |  |  | 0.727 |
| GG | 12 | 10.67(2.93,18.40) | 1 |  | 35.07(-, -) | 1 |  |
| GC+CC | 129 | 10.69(9.29,11.91) | 1.31(0.69,2.50) |  | 28.53(23.46,33.60) | 1.34(0.84,2.06) |  |
| rs3088307 |  |  |  | 0.373 |  |  | 0.23 |
| CC | 103 | 10.23(9.27,11.20) | 1 |  | 28.5(21.61,35.39) | 1 |  |
| GC+GG | 38 | 12.5(10.74,14.26) | 0.82(0.55,1.24) |  | 32.0(27.06,36.94) | 0.68(0.42,1.09) |  |
| rs41430444 |  |  |  | 0.548 |  |  | 0.624 |
| TT | 97 | 10.67(9.56,11.78) | 1 |  | 28.5(20.54,36.46) | 1 |  |
| TC+CC | 44 | 10.43(8.89,11.98) | 1.16(0.77,1.77) |  | 28.63(17.95,39.32) | 0.82(0.52,1.29) |  |
| rs6745710 |  |  |  | 0.376 |  |  | 0.096 |
| CC | 30 | 10.43(9.19,11.68) | 1 |  | 21.17(14.88,27.45) | 1 |  |
| GG+CG | 111 | 10.6(0.18,12.02) | 0.82(0.50,1.33) |  | 30.0(26.47,33.53) | 0.75(0.43,1.31) |  |
| rs2020854 |  |  |  | 0.999 |  |  | 0.553 |
| TT | 127 | 10.6(9.50,11.70) | 1 |  | 28.53(23.00,34.07) | 1 |  |
| CT | 14 | 11.57(7.96,15.18) | 1.0(0.55,1.82) |  | 32.07(16.22,47.91) | 0.83(0.42,1.64) |  |
| rs2297136 |  |  |  | **0.0031** |  |  | **0.0233** |
| AA | 97 | 10.8(9.18,12.42) | 1 |  | 31.4(27.74,35.06) | 1 |  |
| AG+GG | 44 | 9.8(7.14,12.46) | 1.79(1.23,2.83) |  | 20.90(18.80,23.00) | 1.73(1.05,2.88) |  |

CI, confidence interval; HR, hazard ratio

† *P* value was based on log-rank test for PFS and OS in the univariate analysis.
